# Supplementary material for: Exploring county-level spatio-temporal patterns in opioid overdose related emergency department visits
Source: PLoS One. 2022 Dec 30;17(12):e0269509. doi: 10.1371/journal.pone.0269509 (PMC9803238; doi:10.1371/journal.pone.0269509)
Supplement: S1 File — (DOCX) [file pone.0269509.s001.docx]

**S1 File.** **Virginia localities combined for EDOOD visit rate calculation**

- Alleghany County and Covington City
- Albemarle County and Charlottesville City
- Augusta County, Staunton City, and Waynesboro City
- Chesterfield County and Colonial Heights City
- Frederick County and Winchester City
- Fairfax County, Fairfax City, and Falls Church City
- Grayson County and Galax City
- Greensville County and Emporia City
- Henry County and Martinsville City
- Montgomery County and Radford City
- Pittsylvania County and Danville City
- Prince George County, Hopewell City, and Petersburg City
- Prince William County, Manassas City, and Manassas Park City
- Roanoke County, Roanoke City, and Salem City
- Rockingham County and Harrisonburg City
- Rockbridge County, Buena Vista City, and Lexington City
- Southampton County and Franklin City
- Washington County and Bristol City
- Wise County and Norton City
